# The effect of a youth mental health service model on access to secondary mental healthcare for young people aged 14–25 years

**DOI:** 10.1192/bjb.2018.70

**Published:** 2019-02

**Authors:** Sarah Maxwell, Obianuju Ugochukwu, Tim Clarke, Brioney Gee, Emmet Clarke, Hope Westgate, Jonathan Wilson, Belinda R Lennox, Ian M Goodyer

**Affiliations:** 1 Children, Families and Young Peoples Services (CFYP), Norfolk and Suffolk NHS Foundation Trust, UK; 2 Norwich Medical School, University of East Anglia, UK; 3 Department of Psychiatry, University of Oxford, Oxford Health NHS Foundation Trust, UK; 4 Department of Psychiatry, University of Cambridge, Cambridgeshire and Peterborough NHS Foundation Trust, UK

**Keywords:** CAMHS, youth mental health, service design, service access

## Abstract

**Aims and method:**

The Norfolk Youth Service was created in 2012 in response to calls to redesign mental health services to better meet the needs of young people. The new service model transcends traditional boundaries by creating a single, ‘youth friendly’ service for young people aged 14–25 years. The aim of this study was to investigate the effect of the transition to this new model on patterns of referral, acceptance and service use. We analysed routinely collected data on young people aged 14–25 years referred for secondary mental healthcare in Norfolk before and after implementation of the youth mental health service. The number of referrals, their age and gender, proportion of referrals accepted and average number of service contacts per referral by age pre- and post-implementation were compared.

**Results:**

Referrals increased by 68% following implementation of the new service model, but the proportion of referrals accepted fell by 27 percentage points. Before implementation of the youth service, there was a clear discrepancy between the peak age of referral and the age of those seen by services. Following implementation, service contacts were more equitable across ages, with no marked discontinuity at age 18 years.

**Clinical implications:**

Our findings suggest that the transformation of services may have succeeded in reducing the ‘cliff edge’ in access to mental health services at the transition to adulthood. However, the sharp rise in referrals and reduction in the proportion of referrals accepted highlights the importance of considering possible unintended consequences of new service models.

**Declaration of interests:**

None.

Adolescence is a high-risk period for the emergence of mental health problems^1^ but mental health service provision for this age group is often inadequate.^2^^–^^4^ Traditional mental health service models bisect the adolescent period, with young people under 18 (or in some cases 16) years seen by child and adolescent mental health services (CAMHS) and those aged 18 years and over by adult mental health services (AMHS). Consequently, there is often a gap in service provision for young people transitioning to adulthood, meaning that young people are unable to access timely and developmentally appropriate support.^5^^,^^6^ In response to these problems, a number of innovative services have emerged that traverse the traditional CAMHS–AMHS divide in an effort to meet the specific needs of young people and reduce the need for disruptive transitions between services.^6^

Norfolk and Suffolk National Health Service Foundation Trust (NSFT) established the Norfolk Youth Service as a pilot in 2012. The pilot service provided mental healthcare for young people aged 14–25 years with severe and complex non-psychotic mental health problems. Following the success of the pilot, the service was expanded to provide staged intervention for all young people aged 14–25 years living in Norfolk and Waveney, replacing the existing traditional CAMHS–AMHS service model. In 2015, Norfolk and Waveney was home to 271 698 children and young people aged under 25 years.^7^ The county is predominantly rural and 86% of residents identify as White British; it has higher rates of looked after children, children in need because of abuse, neglect or family dysfunction, and pupils with behavioural, emotional and social support needs than the average for England.^7^

The new service model was intended to be youth-oriented, non-stigmatising and recovery-focused to maximise access and engagement. The new service kept the same referral criteria, continuing to accept referrals for young people with mental health problems requiring specialist or secondary care interventions. However, young people no longer had to be referred from a CAMHS service into an AMHS service when they reached 18 years old, increasing the continuity of care. The national and local context for this service transformation, and the design of the new service have been described in detail elsewhere.^8^

The empirical evidence of the effect of redesigning mental health services to bridge the CAMHS–AMHS age range on young people's access to mental health services is limited. The aim of this study was to investigate the effect of implementing a youth mental health service model for young people aged 14–25 years, based on patterns of referral, acceptance and service use. The study involved retrospective analysis of service data collected before and after the implementation of the Norfolk Youth Service.

## Method

### Design

**Fig. 1:**
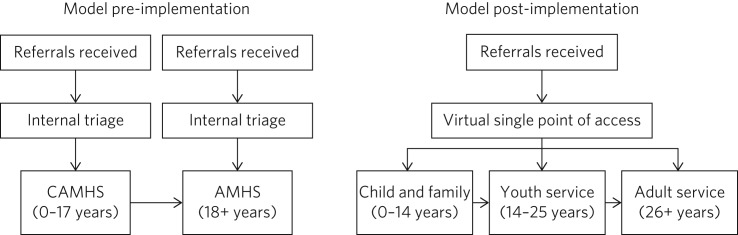
Service models in operation before and after creation of the Norfolk Youth Service as part of Norfolk and Suffolk National Health Service Foundation Trust's redesign of services for children, families and young people. AMHS, Adult Mental Health Services; CAMHS, Child and Adolescent Mental Health Services.

The study used a historical control design. The investigation focused on two 12-month periods, one before and one after the implementation of a specialist youth mental health service for young people aged 14–25 years in Norfolk, UK. The first time period, September 2010 to August 2011, fell immediately before the development of the youth mental health service pilot. The second time period, April 2014 to March 2015, was the second year of operation of the substantive Norfolk Youth Service, chosen to coincide with the completion of the transition to the new service model and embedding of new clinical teams. The service models in operation during these two time periods are outlined in simplified form in Fig. 1. Pre-existing data on young people referred to secondary mental health services in Norfolk during these two time periods was obtained and analysed.

### Data collection

Data on all referrals to NSFT are routinely collected and processed by the Trust's Informatics Department for the purpose of business delivery and development. To meet the aims of our study, the research team requested data held by the Informatics Department on all referrals to secondary mental health services in Norfolk of young people aged 14–25 years during the above 12-month periods. All data was anonymised before being transferred to the research team; the researchers accessed no personally identifiable data. The study was approved by NSFT as a service evaluation and did not require ethical approval.

The data requested included the demographic characteristics of those referred, the outcome of the referral (i.e. whether the young person was accepted into mental health services) and the number of recorded service contacts (i.e. the number of appointments, including both face-to-face and telephone appointments), which served as an indicator of service use.

### Analysis plan

The number of referrals received, proportion of referrals accepted, and the age and gender of those referred and accepted were examined for each of the two time periods under consideration. The average number of service contacts per referral by age was also calculated for each time period. These descriptive statistics were used to make comparisons across the two time periods studied, with the aid of tables and figures. The use of inferential statistics to make comparisons between the two time periods was not considered appropriate given that the dataset included all recorded referrals made during the pre-specified time periods of interest, not a sample of such referrals.

## Results

### Referral and acceptance rates

During a 12-month period before the implementation of the youth service model, from 1 September 2010 to 31 August 2011 inclusive, NSFT received 7476 referrals for young people aged 14–25 years living in Norfolk. Of these referrals, 27.7% were for young people under 18 years of age. Across services, 90.8% of referrals received were accepted. The acceptance rate was higher for AMHS than for CAMHS (95.5 *v.* 78.5%). During a 12-month period post-implementation of the new service model, from 1 April 2014 to 31 March 2015 inclusive, NSFT received 12 559 referrals for individuals aged 14–25 years living in Norfolk. Of these referrals, 45.8% were for young people under 18 years of age. During this period, the Norfolk Youth Service accepted 68.2% of referrals received. The acceptance rate for adults referred to the service was higher than for young people aged under 18 years (75.8 *v.* 59.2%). The referral and acceptance data for both time periods are summarised in Table 1.

**Table 1:** Referrals received and accepted pre- and post-implementation of the youth mental health service model for young people aged 14–17 years and aged 18–25 years

	Pre-implementation			Post-implementation		
	14–17 years	18–25 years	Total	14–17 years	18–25 years	Total
Referred	2070	5406	7496	5746	6812	12 559
Accepted	1624	5162	6786	3400	5163	8563
Percentage accepted	78.5	95.5	90.8	59.2	75.8	68.2

Pre-implementation refers to the 12-month period from 1 September 2010 to 31 August 2011. Post-implementation refers to the 12-month period from 1 April 2014 to 31 March 2015 inclusive.

The majority of those referred during both time periods were female (58.8% pre-implementation and 59.5% post-implementation). For both pre- and post-implementation of the youth service model, the gender disparity in the referrals received was most marked among younger referrals under 18 years of age, with a more even gender split in referrals of those aged over 18 years.

### Service contacts

During the 12-month period before the implementation of the new service model, the total number of contacts with young people aged 14–25 years in Norfolk was 56 759. The average number of contacts per referral was markedly higher for young people aged 15–17 years than for young people aged 18–20 years, with a clear disparity between the rate of contacts per referral for those younger than 18 years and those aged 18 years or over. On average, a young person referred at 17 years of age went on to have 11.3 service contacts, whereas a young person referred at 18 years of age went on to have just 5.3 service contacts.

**Fig. 2:**
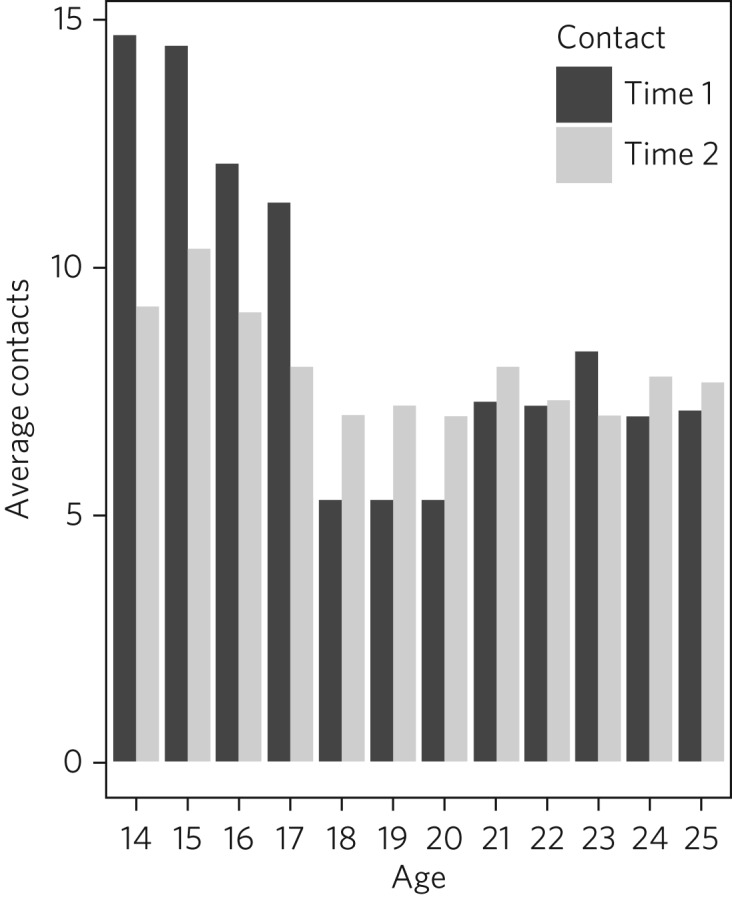
Average number of service contacts per referral for young people aged 14–25 years pre-implementation (Time 1) and post-implementation (Time 2) of the youth mental health service model.

In the 12-month period following implementation of the youth service model, the total number of service contacts with young people aged 14–25 years in Norfolk was 79 659. Although overall the average number of contacts per referral was lower than before implementation of the new model, the average number of contacts received was more equitable across age groups. On average, a young person referred at 17 years of age went on to have eight service contacts, whereas a young person referred at 18 years of age went on to have an average of seven service contacts. The average number of contacts with services during the periods before and after implementation of youth service model by age of referral are presented in Fig. 2.

## Discussion

The purpose of this study was to explore whether a change in service structure from a traditional CAMHS–AMHS model with transition at 18 years of age to a youth mental health service model for young people aged 14–25 years was associated with altered patterns of referral, acceptance or service-use.

A number of interesting changes post-implementation were identified. The number of referrals to mental health services for young people aged 14–25 years living in Norfolk increased by 68% following implementation of the new service model: from 7476 before the implementation of the youth service to 12 559 after its implementation. The number of referrals increased most for young people aged 14–17 years: there was a 2.8-fold increase in the number of individuals aged 14–17 years referred but only a 1.3-fold increase in referrals for individuals aged 18–25 years.

The reason for this increase in referrals is unclear. One possibility is that the increase reflects increased local awareness of the support available for young people with mental health problems as a result of the publicity surrounding the new service model. New referral routes (including the option for young people to self-refer) may also have led to an increased volume of direct referrals to secondary mental health services. Previously, these young people might have been referred to primary care or third-sector agencies in the first instance, with only more severe or complex cases being referred on to secondary care. It is also possible that the increase in referrals reflects a wider increase in demand for mental health support for young people, not directly associated with the change in service model. There is some evidence that rates of internalising problems in children and young people have been increasing in recent years,^9^ and a substantial rise in the demand for children and young people's mental health service has been reported nationally.^10^

Both before and after the implementation of the youth service model, more females were referred than males, with the gender disparity being more marked among younger referrals. The reluctance of young men to seek care for mental health problems is well documented.^11^^,^^12^ The small number of males referred relative to females, both before and after the implementation of the new service model, suggests there is more work to do to encourage young men to access support. NSFT have recently launched a Men's Wellbeing Project that aims to encourage men and boys to talk more openly about their mental health and increase access to mental health services.^13^

Although the raw number of referrals accepted by the service increased substantially following implementation of the youth service model, the proportion of referrals accepted fell: from 91% pre-implementation of the model to 68% post-implementation. This decrease might be at least partially explained by the increased number of referrals coming into conflict with limited service capacity. As previously reported,^8^ a consequence of improving access to services when resources remain limited has been increased wait-lists and sometimes overwhelming case-loads. Although acceptance criteria were unchanged following implementation of the new service model, it is possible that pressures on service capacity might have led to an upward shift in the threshold for secondary care. However, it is also possible that the fall in the proportion of referrals accepted can be explained by an increase in the number of inappropriate referrals due to the introduction of new referral routes. These referrals are then signposted on to more suitable agencies. The service is in the process of investigating this with a view to developing strategies to further improve access for young people across all agencies, to reduce the number of referrals ending up in the wrong place and subsequently being passed around services.

The number of recorded service contacts was used as a proxy for service use. The overall average number of contacts per referral for those aged 14–25 years decreased following the introduction of the youth service model. Although the service offered by Norfolk Youth Service is not time-limited, there is an emphasis on offering appropriately staged intervention and not retaining individuals within the service for longer than needed.^8^ The reduction in overall average service contacts for young people in this age group might, therefore, reflect this change in service philosophy, toward encouraging flexible re-referral if needed.

Before implementation of the Norfolk Youth Service, young people aged 18 years or over were referred to services in high numbers but received substantially fewer contacts with services relative to those aged under 18 years. This ‘cliff edge’ in mental health service use at the transition to adulthood has also been reported in the USA,^14^ suggesting this problem is not specific to the local context. Following the implementation of the youth service model, the average number of contacts per referral was more equitable across ages, with the cliff edge in service contacts no longer evident. Pre-implementation, the average number of contacts per referral at 18 years of age was less than half that at 17 years of age. Post-implementation, the average number of contacts per referral was just one fewer for 18-year-olds than for 17-year-olds.

It is possible that the new service model simply moved the transition down from 18 years to 14 years. Data for 13-year-olds demonstrated that this was not the case: 574 referrals for 13-year-olds were accepted by the service and they received 5103 contacts. This gave them an average of 8.9 contacts per referral, which is broadly similar to 14-year-olds. This does not support the idea that the previous disparity in contacts per referral has been moved to a transition at 14 years instead of 18 years.

Overall, this study suggests that implementation of the youth service model might have been successful in reducing the disparity between demand for, and access to, service during young adulthood.

### Limitations

As the study had a historical control design, it is not possible to know whether the changes in referral, acceptance and service-use patterns observed following implementation of the youth service model were the result of the change in service model. It remains possible that the changes observed resulted from wider factors influencing demand for and/or engagement with mental health services. Further, because the data used were routinely collected service data, it is possible that there were variations in the quality of data collection over time that may have affected the study's findings.

Although moving from separate CAMHS and AMHS to an integrated service for 14- to 25-year-olds removes the service boundary at age 17/18 years, it also creates new boundaries at age 13/14 years and 25/26 years. Arguably, these new service boundaries fit more closely with developmental transitions and coincide less closely with peaks in the incidence of mental health problems. Nonetheless, it will be important for future research to investigate the effect of these new service boundaries on those who fall outside the 14–25 year age range.

## About the authors

**Sarah Maxwell** is a consultant child and adolescent psychiatrist with Children, Families and Young Peoples Services (CFYP), Norfolk and Suffolk NHS Foundation Trust, UK. **Obianuju Ugochukwu** is a consultant general adult psychiatrist with Children, Families and Young Peoples Services (CFYP), Norfolk and Suffolk NHS Foundation Trust, UK. **Tim Clarke** is a research clinical psychologist with Children, Families and Young Peoples Services (CFYP), Norfolk and Suffolk NHS Foundation Trust and Norwich Medical School, University of East Anglia, UK. **Brioney Gee** is a research psychologist with Children, Families and Young Peoples Services (CFYP), Norfolk and Suffolk NHS Foundation Trust and Norwich Medical School, University of East Anglia, UK. **Emmet Clarke** is an assistant psychologist with Children, Families and Young Peoples Services (CFYP), Norfolk and Suffolk NHS Foundation Trust, UK. **Hope Westgate** is an assistant psychologist with Children, Families and Young Peoples Services (CFYP), Norfolk and Suffolk NHS Foundation Trust, UK. **Jonathan Wilson** is a research director at Children, Families and Young Peoples Services (CFYP), Norfolk and Suffolk NHS Foundation Trust and Norwich Medical School, University of East Anglia, UK. **Belinda R Lennox** is an associate professor and clinical senior lecturer in the Department of Psychiatry, University of Oxford, Oxford Health NHS Foundation Trust, UK. **Ian M Goodyer** is a professor emeritus in the Department of Psychiatry, University of Cambridge and Peterborough NHS Foundation Trust, UK.
